# Finding the right balance against bioterrorism.

**DOI:** 10.3201/eid0504.990405

**Published:** 1999

**Authors:** R A Clarke

**Affiliations:** The White House, Washington, DC, USA.


### Finding the Right Balance against Bioterrorism

**Richard A. Clarke**
The White House, Washington, D.C., USA

For the first time the Deparment of Health and Human Services is part of the national security apparatus of the United States. That reflects a change in our views on chemical and biological defense programs. Almost 5 years ago at the bidding of the president we began to look at what has come to be known as "asymmetrical threats," ways in which opponents (be they nations or terrorist groups) could attack us without directly engaging our military forces. At the same time we were faced with two events that drew our attention to chemical and biological threats. Iraq used chemical weapons on Iran and on its own citizens and appeared to be concealing a biological weapons program. Also, the hitherto unknown Japanese cult Aum Shinrikyo used sarin nerve agent in the Tokyo subway; the cult failed in an attempt to use biological weapons against Americans in Japan.

In 1998, the president launched the first national effort to create a biological weapons defense for the United States. While some believe that the response is not strong enough, many others think that the proposed program exaggerates the threat, that biological weapons are too unpredictable, and that the only big biological weapons program died with the Soviet Union. However, the former Soviet Union was not the only state engaged in biological weapons research and development. Almost every nation on the State Department's list of nations that sponsor terrorism has engaged in chemical and or biological weapons development. If these nations have armed, trained, funded, and advised terrorist groups, they could cross the line and provide terrorists with chemical or biological weapons. Finally, some critics say that until we really know about a specific threat to use these weapons against the United States, we should not be raising the specter of horror; instead we should be quietly working in Geneva to improve the ban on biological weapons. We are pushing in Geneva, but that is not enough. When we learn of a specific threat, it will be too late to do research and development, too late to procure medicines, too late to train local authorities.

The current bioterrorism initiative includes a new concept: the first-ever procurement of specialized medicines for a national civilian protection stockpile. As new vaccines and medicines are developed, that program can be expanded. The initiative includes invigoration of research and development in the science of biodefense; it invests in pathogen genome sequencing, new vaccine research, new therapeutics research, and development of improved detection and diagnostic systems. The 2-year program provides for Department of Health and Human Services research, almost tripling the previous 2-year effort, in addition to ongoing work in the Defense Department, and it includes a reinitiation of the federal program to help state and local public health infrastructure and surveillance systems.

The biological weapons protection program is part of the overall chemical and biological protection effort, which includes aid to state and local governments for first-responder training, planning, exercises, and equipment.

Richard A. Clarke serves as the country's first National Coordinator for Security, Infrastructure Protection and Counter-Terrorism. He was Deputy Assistant Secretary of State for Intelligence in the Reagan Administration and served in the Bush Administration as Assistant Secretary of State for Politico-Military Affairs.

Address for correspondence: Richard A. Clarke, National Security Council, 17th and Pennsylvania Avenue, N.W., Washington, D.C. 20504; fax: 202-456-9360.

